# Terrible Triad of the Shoulder: A Case Series and Literature Review

**DOI:** 10.7759/cureus.47699

**Published:** 2023-10-26

**Authors:** Zinon Kokkalis, Vasileios Giannatos, Spyridon Papagiannis, Antonis Kouzelis, Andreas Panagopoulos

**Affiliations:** 1 Orthopedics and Traumatology, University General Hospital of Patras, Patras, GRC

**Keywords:** motorvehicle accident, shoulder dislocation, brachial plexus injury, shoulder, shoulder terrible triad

## Abstract

Introduction:The shoulder terrible triad is an underdiagnosed injury pattern consisting of anterior shoulder dislocation, rotator cuff tear, and nerve injury from the brachial plexus in its original description. The purpose of this study is to raise awareness of the condition, suggest treatment strategies, and emphasize the difficulties in treating this condition.

Methods:This case series of seven patients from the same institution. All patients underwent x-rays before and after the reduction of the dislocation, MRI to assess the musculoskeletal injuries, and EMG and clinical examination to assess the nerve lesions. Early arthroscopic repair was opted for the rotator cuff tears. A conservative approach was chosen for the nerve lesions. Active forward flexion and external rotation, Constant score, and Visual to Analogue Scale (VAS) were recorded pre- and post-operatively.

Results: All the patients showed an improvement in function postoperatively. However, four of the seven patients did not recover fully. The mean Constant and VAS scores were improved from 15.2 +/- 2.8 (12 to 19) to 67 +/- 16.6 (44 to 86) and from 7.5 +/- 1 (6 to 9) to 2.3 +/- 0.8 (1 to 3), respectively. The patients were followed up for a mean time of 28.2 +/- 10.1 months (18 to 43 months). Time-to-surgery shorter than four weeks showed better results, but not statistically significant.

Conclusions:The diagnosis of the shoulder terrible triad requires a high level of suspicion. Early arthroscopic repair for the rotator cuff tears and waiting for the nerve recovery is suggested. Delayed time from injury to surgery might be related to worse outcomes, but higher-level research is needed in this direction.

## Introduction

The shoulder is the most commonly dislocated joint (45% of all dislocations) faced by emergency physicians. More than 95% are anterior dislocations, the mechanism is most commonly excessive external rotation (ER) with abduction or hyperextension in the overhead direction [1]. According to the same study with a pool of 240 patients, assessed with MRI and U/S, rotator cuff tear was seen on 27.91% of the patients after anterior shoulder dislocation, axillary nerve injury was seen in 15.83%, 20.83% showed a Bankart lesion and 15.42% sustained a greater tuberosity fracture [1]. The age distribution is bimodal, with the largest group being young adult men with high-energy injuries and the second group older patients with low-energy trauma [2].

The “shoulder terrible triad’’ refers to a rare type of injury consisting of anterior shoulder dislocation accompanied by a rotator cuff tear and a brachial plexus injury. The injury was first reported by Neviaser et al. in 1988 [3]. Gonzalez et al. [4] and Güven et al. [5] described the injury as the “unhappy triad’’ and in 1995, Groh et al. first used the name “shoulder terrible triad’’ [6]. Since then, few studies, mostly case reports and case series, have been published, describing the injury pattern and the methods of treatment [7-16]. A recent retrospective cohort study by Marsalli et al. attempted to classify the shoulder terrible triad, expanding the definition. According to them, a shoulder terrible triad consists of an abductor injury (rotator cuff or greater tuberosity), a brachial plexus injury (distal nerves or shoulder nerves) and an anterior glenoid injury (capsulolabral or bony Bankart) [10].

The primary objective of this study is to describe the functional outcomes and the effect of time of presentation to surgical intervention in patients presenting with the “shoulder terrible triad,’’ review the current treatment strategies and raise awareness regarding the difficulties of treating such injuries.

## Materials and methods

Seven patients (five men and two women) were treated in the Shoulder and Elbow Department of the Patras University Hospital from March 22, 2018 to July 12, 2020 (Table 1). The mean age of the patients was 61.6 +/- 7.6 years old (51 to 74). Five of them had a right anterior shoulder dislocation and two faced a left anterior shoulder dislocation. A closed reduction under sedation was performed on all the patients. X-rays were performed before and after the reduction in each patient. A clinical examination and an EMG were performed on all the patients, according to which a diagnosis of the brachial plexus injury was reached. An MRI was also conducted after the closed reduction but preoperatively in order to assist the brachial plexus injury diagnosis as well as the soft tissue injuries. All patients underwent an arthroscopic rotator cuff repair by the same surgeon after a mean time of 7.9 +/- 15.8 weeks from the injury (2 to 22). The beach chair position was used, due to its quick conversion to an anterior open approach and the traction nerve injuries associated with the lateral decubitus position [17,18]. Large tears with full rupture of the supraspinatus and infraspinatus were diagnosed in all of the patients. All rotator cuff tears were repaired using a double-row technique (medial and lateral columns) with a mean number of five anchors [3-6]. A concomitant Bankart lesion was found in some patients, but no Bankart repair was performed, in order to avoid shoulder stiffness. For the neurological injuries, a conservative approach and observation were chosen. After the surgery, all patients were immobilized in 45º shoulder abduction using an abduction pillow. The evaluation of the patients was performed preoperatively and postoperatively before discharge, using clinical examination, the Constant Score translated in Grintok, the Visual to Analogue Scale (VAS), and the active range of motion of Forward Flexion (FF) and ER [19].

**Table 1:** Patient data SH: Shoulder, INJ TO SUR: Time interval from time of injury to surgery (measured in weeks), BPI: Brachial Plexus Injury, Pre-op: Pre-operatively, Post-op: Post-operatively, FF: Forward Flexion, ER: External Rotation, VAS: Visual to Analogue Scale, F-UP: Follow-Up, post cord: posterior cord, lat cord: lateral cord, ax cord: axillary cord

Patient No	SEX	AGE	SH	INJ TO SUR (wks)	BPI lesion	Pre-op				F-UP	Post-op				Re to work	Complications
						VAS	Constant	FF	ER		VAS	Constant	FF	ER		
1	M	61	R	2	post cord - ax	8	12	30	10	18	3	56	70	20	no	Partial re-rupture
2	M	66	R	6	post cord - ax	7	15	20	0	43	3	58	60	30	no	
3	F	54	R	4	lat cord - ax	6	19	30	10	18	1	80	140	60	y	
4	M	64	R	2	post cord -radial	7	17	30	0	36	2	86	160	80	y	
5	F	51	L	3	post & lat cord - ax	9	16	60	10	24	3	78	160	60	y	
6	M	61	L	16	post cord - ax	8	12	20	0	30	2	44	30	10	no	Re-rupture
7	M	74	R	22	ax	6	18	30	9	24	1	64	110	10	no	

A literature review was performed in PubMed using the terms “Shoulder terrible triad’’ and 67 results were returned. All articles were reviewed, and 17 case reports and case series were found describing a shoulder terrible triad injury. The articles were extensively analyzed, and common injury patterns and treatment practices were recognized, to compare with our own treatment plans. Statistical analysis was performed on the preoperative and postoperative values of Constant score, VAS, FF, and ER. The mean values and standard deviation of age and time to surgery were also calculated. SPSS (IBM Corp., Armonk, NY) was used for the above calculations of mean values and standard deviation as well as the bivariate correlation of pre-operative and post-operative scores using the Pearson coefficient. Time to surgery shorter than four weeks was compared to the time of surgery longer than four weeks using the non-parametrical two independent variables test, Mann-Whitney U test. A P-value of <0.05 was considered statistically significant for this case series.

## Results

**Figure 1:**
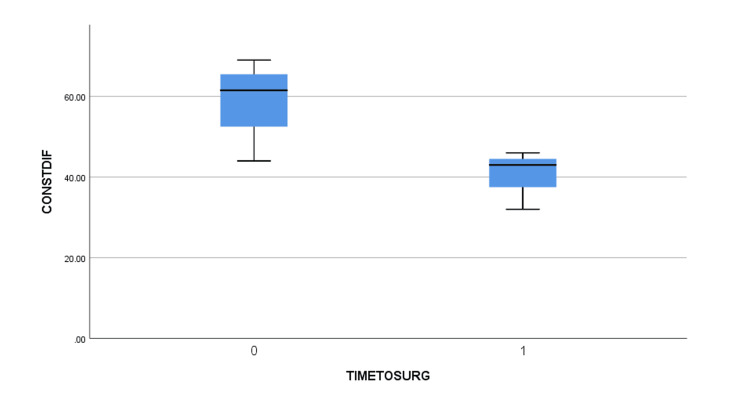
Pre-operatively and post-operatively constant difference between early (TIMETOSURG=0) and late (>4 weeks, TIMETOSURG=1) surgical intervention groups. CONSTDIF = Post-operatively Constant Score – Pre-operatively Constant Score, TIMETOSURG = Time interval from injury to surgery, 0 for <4 weeks, 1 for >4 weeks.

**Figure 2:**
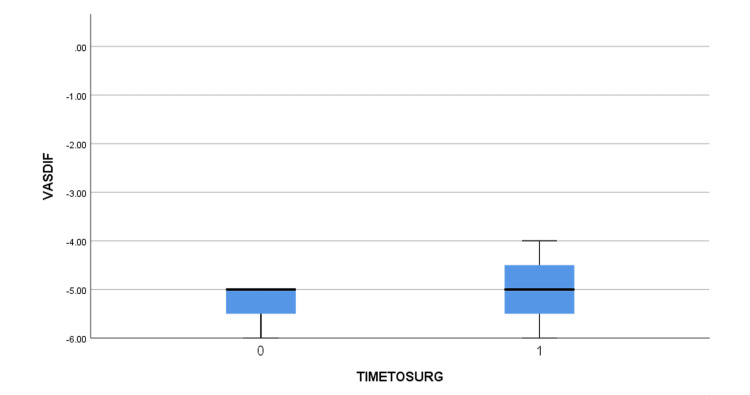
Pre-operatively and post-operatively VAS difference between early (TIMETOSURG=0) and late (>4 weeks, TIMETOSURG=1) surgical intervention groups. VASDIF = Post-operatively VAS – Pre-operatively VAS, TIMETOSURG = Time interval from injury to surgery, 0 for <4 weeks, 1 for >4 weeks.

**Figure 3:**
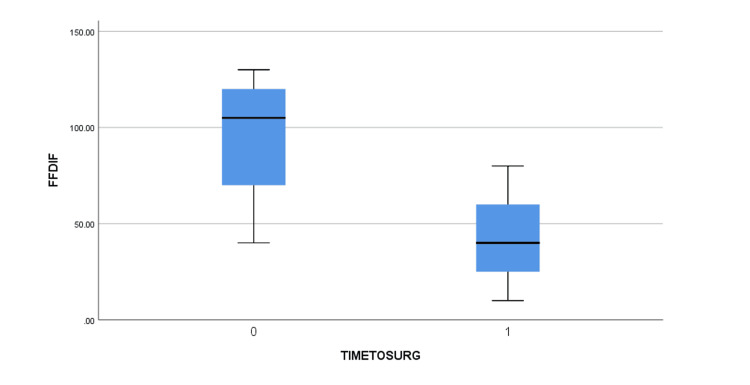
Pre-operatively and post-operatively forward flexion difference between early (TIMETOSURG=0) and late (>4 weeks, TIMETOSURG=1) surgical intervention groups. FFDIF (in ^o^) = Post-operatively Forward Flexion – Pre-operatively Forward Flexion, TIMETOSURG = Time interval from injury to surgery, 0 for <4 weeks, 1 for >4 weeks.

**Figure 4:**
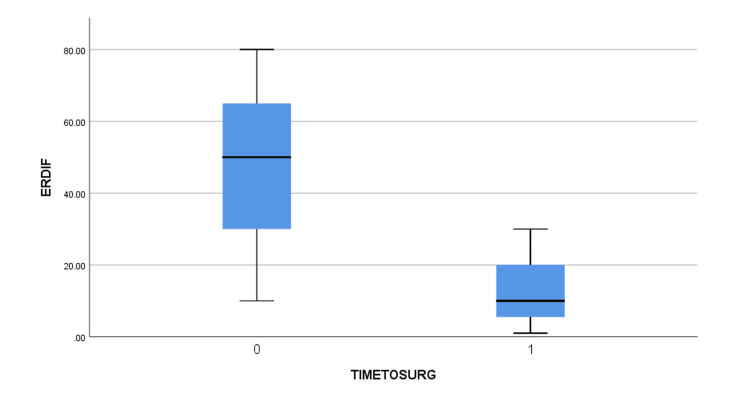
Pre-operatively and post-operatively external rotation difference between early (TIMETOSURG=0) and late (>4 weeks, TIMETOSURG=1) surgical intervention groups. ERDIF (in ^o^) = Post-operatively External Rotation – Pre-operatively External Rotation, TIMETOSURG = Time interval from injury to surgery, 0 for <4 weeks, 1 for >4 weeks.

The mean age of the patients was 61.6 +/- 7.6 years old (51 to 74) (Table 2). Five of them had a right anterior shoulder dislocation and two faced a left anterior shoulder dislocation. Using the clinical findings and the EMG, the brachial plexus injuries were assessed. Three of the patients were diagnosed with a posterior cord lesion (abduction and ER weakness along with a weakness of elbow extension) with a more profound axillary lesion (abduction and ER weakness were more evident than the extension weakness). One patient presented with a lateral cord injury (weakness of elbow flexion and supination) and a more evident axillary lesion. One patient presented with a combined posterior and lateral cord injury, again with the axillary lesion being the primary deficit. Another patient was diagnosed with a posterior cord injury with a major lesion of the radial nerve (elbow extension weakness was more prominent). Finally, one patient suffered solely an axillary nerve lesion. The MRI showed large rotator cuff tears with full rupture of the supraspinatus and infraspinatus, Bankart lesion in some of the patients but no Greater Tuberosity fractures were detected. Preoperatively, all patients experienced a shoulder palsy with no active movement. The arthroscopic repair was performed by the same surgeon in all patients after a mean time of 7.9 +/- 15.8 weeks from the injury (2 to 22). The patients were followed up postoperatively for a mean time of 27.6 +/- 18.6 months (18 to 43 months). Full restoration of function was not achieved in all the patients, with four out of seven showing a lower Constant score than the others. The mean Constant and VAS scores were improved from 15.6 +/- 5.6 (12 to 19) to 66.8 +/- 30.4 (44 to 86) and from 7.29 +/- 2.2 (6 to 9) to 2.1 +/- 1.8 (1 to 3), respectively. Similarly, the active range of motion of FF and ER was greatly improved before discharge from 31.4^o^+/- 26.8^o^ (20^o^ to 60^o^) and 5.6^o^ +/- 10.4^o^ (0^o^ to 10^o^) to 104.3^o^ +/- 103,8^o^ (30^o^ to 160^o^) and 38.6^o^ +/- 55.8^o^ (10^o^ to 80^o^). No instability was noted during the last follow-up. After the bivariate correlation using the Pearson coefficient was performed, the VAS and Constant scores were found to be statistically significant pre- and post-operatively with p=0.037 and p=0.04, respectively. Additionally, the Mann-Whitney U test showed no statistically significant difference between pre- and post-operatively differences between the early and late (>4 weeks) surgical intervention groups, but some tendency was noted, and the difference is evident, as seen in the boxplots (Figures 1-4).

**Table 2:** Comparison of pre-operative and post-operative constant, visual to analogue scale, forward flexion, and external rotation values FF: Forward Flexion, ER: External Rotation, VAS: Visual to Analogue Scale, op: operatively

	Mean +/- 2SD	P-value
Pre-op FF	31.4+/- 26.8	P=0.092
Post-op FF	104.3 +/- 103.8	
Pre-op ER	5.6 +/- 10.4	P=0.972
Post-op ER	38.6 +/-55.8	
Pre-op Constant	15.6 +/- 5.6	P=0.04
Post-op Constant	66.8 +/- 30.4	
Pre-op VAS	7.29 +/- 2.2	P=0.037
Post-op VAS	2.1 +/- 1.8	

## Discussion

The shoulder terrible triad is a rare injury, most commonly presenting as a rotator cuff tear with anterior capsular injury and axillary nerve injury. Other patterns can also be observed, such as a radial nerve injury, a bony Bankart injury, or a greater tuberosity, as described in a classification proposed by Marsalli et al. in 2020 [10]. Although rare, the orthopedic surgeon must retain a high level of suspicion, especially after persisting pain and weakness or inability to abduct after shoulder relocation [3-4,6]. Care should be taken to differentiate the inferior shoulder dislocation from similar concomitant injuries, as this suggests Luxatio Erecta Humeri, a different clinical entity [20]. The increasing literature reports through the years imply that the shoulder terrible triad injury pattern was probably under-documented and recent literature reports raised suspicion and contributed to their recognition. Its treatment remains challenging to this day. According to our results above, both the Constant and VAS scores were significantly improved after the surgery and before the discharge (p=0.04 and p=0.037), but the improvement of the clinical examination was evident as well. For both the VAS and Constant scores and the FF and ER active range of motion, we observed two groups of patients, three patients with great outcomes and four patients who did not achieve full recovery. Four out of the seven patients, the ones who did not achieve full recovery, did not manage to return to work, the reason being re-rupture with decline for re-operation, concomitant more serious pathologies, or pension. A tendency for better outcomes when patients are operated on in less than four weeks was noted but not statistically confirmed, perhaps due to the small sample of this case series. Two patients experienced re-rupture, they were offered surgery, but it was declined. Clinically, both patients experienced limited active ROM regarding shoulder abduction and ER, with no pain experienced, however. All patients were greatly improved neurologically, including the deltoid muscle innervation as evaluated by functional tests for the deltoid. The functional deficits post-surgically in these three patients were attributed mainly to the musculoskeletal injuries.

Nerve lesions

In all the articles reviewed, as well as our own cases, a conservative approach was chosen for neurological injuries, with good results one year later more than half of the time [13]. Kokkalis et al. in 2017 provided a review of the brachial plexus and axillary nerve injuries and summarized the mechanisms of nerve injury as direct pressure, repetitive microtrauma, compression, and stretch-induced ischemia [21]. Seddon et al. classified nerve injuries as neuroapraxia, axonotmesis, and neurotmesis, with neurotmesis leading to complete functional loss and the need for surgical intervention [21,22]. Specifically for the shoulder terrible triad injury pattern, Strafun et al. proposed a conservative approach, especially for the nerve injuries preserving 30% of nerve conduction during preoperative EMG [23]. One mechanism of nerve injury proposed is the crushing of the humoral head in the axillary border, but the mechanism of traction of the brachial plexus and distal nerves prevails, with the position of the limb during the accident affecting the nerves injured [24]. The infraclavicular roots of the posterior cord are the most often injured, including the axillary (it follows a short, fixed course in the quadruple space and around the surgical neck of the humerus) and radial nerves and usually corresponds to neuropraxia (three months recovery) or axonotmesis (six to seven months recovery) [9,10,21]. Many authors propose surgical treatment when there is no clinical or EMG evidence of recovery after three or four months, but newer research proposes longer waiting times before surgical interventions, as nerve lesions after shoulder dislocations seem to still recover months later with a success rate of 87.5% to 100%, especially for infraclavicular lesions in contrast to supraclavicular [10,21,24-26]. Emergency surgical indications in general consist of vascular injury or severe pain/muscle paralysis, with the treatment options being nerve transfers, nerve grafting, muscle transfers, and neurolysis, nerve transfers being the most successful [21]. Poor prognostic factors have been recognized such as high age, the presence of hematoma, and the longer time period between dislocation and reduction [24]. More distal nerve lesions are associated with better outcomes [10].

Rotator cuff tears

Absence of shoulder abduction after the reduction is mainly due to the rotator cuff tear. Axillary nerve injury may cause abduction weakness but the inability to do so suggests RCT. The surgical approach is the go-to treatment for traumatic RCT, especially in younger individuals, in order to avoid muscle atrophy [10,27]. Although a Bankart repair is suggested by the literature after first-time dislocations, we performed only RC repair and no Bankart repair as in our experience the problem seems to be the shoulder stiffness postoperatively and not instability [10]. A Bankart repair would only increase the stiffness postoperatively. No redislocations were recorded. The surgical approach for the RCT produced statistically significant results regarding the Constant and VAS scores compared to preoperative scores in our case series. However, there is room for improvement, as two of our seven patients experienced a re-rupture, despite the double-row suturing technique. Both patients presented with painless ROM but limited ER and abduction after the re-tear. A study by Marsalli et al. found the incidence of retears at 24%, which is in agreement with our results [10].

Outcomes

As concluded from our results, a shoulder terrible triad is a difficult injury pattern. Three of our patients had a favorable outcome but four experienced reduced functionalities during our follow-up. Time to surgery might play a role in the outcome, as three of the four patients with the persisting disabilities were operated on 22, 16, and six weeks after the injury. In contrast, patients who were operated on two or three weeks after the injury showed more favorable outcomes. However, more and higher evidence trials would be needed to establish such a connection, as the number of patients in this trial was limited in order to draw conclusions.

## Conclusions

A high level of suspicion is needed to recognize the shoulder's terrible triad injury pattern. A conservative approach is opted for most of the time for nerve injuries, whereas a surgical approach encompasses good outcomes for rotator cuff tears. Our current treatment plan does not guarantee a favorable outcome in all cases, mainly due to the poor quality of the torn rotator cuff. Increased time from injury to operation might be related to worse outcomes, but a higher level of evidence is needed in this direction to establish the relation.
